# Hepatic Arterial Infusion Chemotherapy With Folfirinox or Oxaliplatin Alone in Metastatic Colorectal Cancer

**DOI:** 10.3389/fmed.2022.830595

**Published:** 2022-06-16

**Authors:** Violaine Randrian, Simon Pernot, Baptiste Sionneau, Denis Smith, Annie Lim, Yann Touchefeu, Claire Gallois, Anthony Turpin, Sahir Javed, Rosine Guimbaud, Pascale Rivera, Mehdi Karoui, Edouard Auclin, Julien Taieb

**Affiliations:** ^1^Department of Hepato-Gastro-Enterology, CHU Poitiers, Poitiers, France; ^2^Department of Medical Oncology, Institut Bergonié, Bordeaux, France; ^3^Medical Oncology, Bordeaux University Hospital, Bordeaux, France; ^4^Department of Gastroenterology, Clinique Santé Atlantique, Saint-Herblain, France; ^5^Department of Hepatogastroenterology, Digestive Oncology, Nantes University Hospital, Nantes, France; ^6^Department of Gastrointestinal Oncology, Université de Paris, Hôpital Européen Georges Pompidou, Paris, France; ^7^Department of Oncology, CHU Lille, Lille, France; ^8^Department of Digestive Oncology, IUCT-Rangueil, CHU Toulouse, Toulouse, France; ^9^Department of Surgical Oncology, Université de Paris, Hôpital Européen Georges Pompidou, Paris, France; ^10^Methodology and Quality of Life Unit in Oncology, University Hospital of Besançon, Bourgogne Franche-Comté University, INSERM, EFS BFC, UMR 1098, Interactions Hôte-Greffon-Tumeur/Ingénierie Cellulaire et Génique, Besançon, France; ^11^Department of Medical Oncology, Université de Paris, Hôpital Européen Georges Pompidou, Paris, France

**Keywords:** colorectal cancer, hepatic arterial infusion, liver metastases, cancer treatment, surgery

## Abstract

**Background:**

Hepatic arterial infusion (HAI) of chemotherapy is an option for the treatment of patients with liver metastases from colorectal cancer (LMCRC). Though HAI with oxaliplatin (HAI-Ox) is generally used, intravenous (IV) 5-fluoro-uracil (5FU)-oxaliplatin-irinotecan HAI (HAI-Folfirinox) is feasible and leads to curative-intent surgery in 30% of pretreated patients. We compared the efficacy and safety of HAI-Ox and HAI-Folfirinox.

**Methods:**

Patients who underwent HAI chemotherapy for LMCRC were retrospectively included from 2008 to 2019 from six French expert centers.

**Results:**

Data were collected from 273 previously treated patients with LMCRC. Patients received HAI-Folfirinox (*n* = 52) or HAI-Ox (*n* = 221) combined with IV chemotherapy. The objective response rate (ORR) was 43.2% in patients with HAI-Folfirinox and 45.9% (ns) in patients with HAI-Ox. Median overall survival (OS) was 17 months (95% CI: 15–32.3) with HAI-Folfirinox and 26.2 months (95% CI: 19.4–34.4; *p* = 0.1) with HAI-Ox. Median progression-free survival (PFS) was 7.9 months (95% CI: 4.9–10.3) with HAI-Folfirinox and 6.4 months (95% CI: 6.0–7.7; *p* = 0.6) with HAI-Ox. The secondary liver resection rate was 35.6% with HAI-Folfirinox and 16.7% with HAI-Ox (*p* = 0.007). Grade 2 and above toxicities were significantly more frequent with HAI-Folfirinox. In the global population, only 2 factors were prognostic for OS in multivariable analyses: liver-only disease [hazard ratio (HR): 0.4; 95% CI 0.20–0.83; *p* = 0.013] and local complications of the catheter (HR: 3.8; 95% CI 1.6–9.0; *p* = 0.002).

**Conclusion:**

Hepatic arterial infusion results in high response rates, secondary resections, and long survival in pretreated patients with LMCRC.

## Introduction

Colorectal cancer (CRC) management raises new challenges as its incidence increases worldwide. As the first metastatic site, hepatic progression contributes to death in half of the patients with metastatic disease (1). This trend has prompted the medical community to elaborate more aggressive systemic and loco-regional approaches even after several lines of treatment (2, 3). Although hepatic arterial infusion (HAI) chemotherapy relies on a robust biological rationale, its development has been initially restricted by two limitations: first, the complexity of surgical removal of the catheter with a significant local complications rate and, second, extra-hepatic progressive lesions excluding patients from an HAI loco-regional treatment approach (4). With technical advances in catheter insertion through interventional radiology procedures (5), HAI chemotherapy has become an effective option for the treatment of patients with liver-dominant/exclusive disease. Moreover, the global strategy for the management of these patients has evolved, and HAI is now mostly used in combination with active systemic treatment, allowing better control of extrahepatic disease when limited (4).

Hepatic arterial infusion with oxaliplatin combined with intravenous (IV) 5-fluoro-uracil (5FU) has yielded positive results in patients harboring CRC liver metastases (6–8) (LMCRC), with a 16% secondary surgery rate (9). These preliminary results led to ongoing randomized phase III trials assessing HAI-Ox in the first- and second-line settings in non-resectable LMCRC (OSCAR NCT02885753, SULTAN NCT03164655) or in the adjuvant setting after liver surgery (PACHA-01, NCT02494973) (10).

The multicenter phase II trial, OPTILIV (11) has evaluated an intensified regimen with 5FU, oxaliplatin, and irinotecan administered intra-arterially combined with IV cetuximab in *RAS* wild-type patients with liver metastases from CRC after the failure of IV chemotherapy (12). About 30% of these heavily pretreated patients finally underwent hepatic resection. This regimen was associated with non-negligible grade 3–4 toxicities, in particular neutropenia (42.6%). To our knowledge, there is no study comparing HAI with oxaliplatin (HAI-Ox) and HAI with Folfirinox (HAI-Folfirinox) in patients with non-resectable LMCRC. In this multicenter retrospective study, we looked at HAI performance in pretreated patients with liver-dominant/exclusive mCRC and compared IV chemotherapy or targeted therapy combined with HAI-Ox or with HAI-Folfirinox. We assessed the efficacy and safety of these administration modalities in patients with disease refractory to standard therapies.

## Materials and Methods

### Patients

Patients who underwent HAI chemotherapy for liver metastases from pathologically proven colorectal cancer refractory to at least one standard IV regimen were retrospectively included from 2008 to 2019.

Data were collected from routinely treated patients with HAI chemotherapy of six expert centers.

The study was performed according to the principles of the Declaration of Helsinki.

### Treatments

Hepatic arterial infusion treatment was delivered through a catheter, which was implanted percutaneously by an interventional radiologist. This catheter was linked to an implantable port, intra-arterial catheter patency and hepatic artery skeletonization or occlusion were checked by contrast medium opacification before treatment initiation and every two courses of treatment. HAI-Ox was delivered at the dose of 85 mg/m^2^ (86.5%) or 100 mg/m^2^ (13.5%) in 2 h and IV chemotherapy with or without targeted therapy according to *RAS* status (bevacizumab or cetuximab). HAI-Folfirinox was based on the OPTILIV study protocol (11) as follows: 5-FU 2,800 mg/m^2^, irinotecan 180 mg/m^2^, and oxaliplatin 85 mg/m^2^. HAI-Folfirinox was administered alone or in association with IV bevacizumab or cetuximab according to *RAS* status.

### Data Collected

Data were collected retrospectively from electronic medical records. Included were demographic data (age and gender), disease characteristics (date of diagnosis of colorectal cancer and metastatic status, sites of metastases, number and distribution of liver metastases, and WHO PS), molecular data (*KRAS*, *RAS*, *BRAF*, and microsatellite instability status), laboratory parameters (bilirubin, ACE CA19-9 before treatment and during follow-up), previous treatments (surgical resection, radio-ablation, and previous systemic treatment lines), concomitant anti-cancer treatment (IV chemotherapy and targeted therapies), tolerability data (treatment-related toxicities, dose reductions, and treatment stop), and response to treatment and survival (objective hepatic and extra-hepatic response, date of hepatic and extra-hepatic progressions, last date of follow-up, and status at last follow-up).

### Statistical Analysis

Progression-free survival (PFS) was defined as the time elapsed from the beginning of HAI treatment until progression (any site for global-PFS, hepatic for liver-PFS) or death (all causes). Overall survival (OS) was defined as the time elapsed from the beginning of HAI treatment until death (all causes).

Survival curves were estimated using the Kaplan-Meier method. Median follow-up and the 95% confidence intervals (CI) were calculated using the reverse Kaplan-Meier method.

The statistical association of patients with tumor-related characteristics and treatment with survival was first assessed by the univariate Cox proportional hazards model. Parameters with values of *p* < 0.10 in univariate analysis and/or clinically relevant variables were entered into the multivariable Cox-regression model. Values of *p* < 0.05 were considered statistically significant. All analyses were performed using R software version 2.15.2 (R Development Core Team, Vienna, Austria).

## Results

### Patient Characteristics

We included 273 patients previously treated by one or more chemotherapy protocols (Supplementary Table 1). Among them, 52 (19.0%) received HAI-Folfirinox and 221 (81.0%) received HAI-Ox. The tumor molecular profile was only available for a subset of patients (109/273; 39.9%) according to the study period. *KRAS* status was available in 39.2% of patients. *KRAS* mutations were similar in the HAI-Folfirinox and HAI-Ox groups (31.6% vs. 43.0%; *p* = 0.124). *BRAF* mutations represented 7.4% of the 122 tumors analyzed and microsatellite instability represented 7.3% of the 69 tumors analyzed. The disease was liver-limited in 69.5% of patients and liver-dominant in the remaining 30.5%. Peritoneal, lung, and lymph node metastatic lesions were described in 5.6, 20.7, and 4.2% of patients, respectively, with no significant difference between the two groups (Table 1). All patients were previously treated with 5-FU. Most patients had previously received irinotecan (231/273; 84.9%) and oxaliplatin (231/273; 84.9%). HAI chemotherapy was proposed as a second-line or third-line treatment in 42.9 and 32.2% of cases, respectively.

**TABLE 1:** Characteristics of the extra-hepatic disease in the population.

Hepatic intra-arterial treatment	All patients *N* = 273	HAI-Folfirinox *N* = 52	HAI-Oxaliplatin *N* = 221	*p*
**Peritoneal metastases**				0.605
Yes	12 (5.6%)	0 (0.0%)	12 (6.2%)	
No	201 (94.4%)	18 (100.0%)	183 (93.9%)	
Unknown	60	34	26	
**Lung metastases**				1
Yes	9 (4.2%)	3 (16.7%)	41 (21.0%)	
No	204 (95.8%)	15 (83.3%)	154 (79.0%)	
Unknown	60	34	26	
**Lymph node metastases**				1
Yes	9 (4.2%)	0 (0.0%)	9 (4.6%)	
No	204 (95.8%)	18 (100.0%)	186 (95.4%)	
Unknown	52	34	26	

### Treatment

Patients in the HAI-Folfirinox group received a median of 4 cycles (range: 1–27) and patients in the HAI-Ox group received a median of 5 cycles (range: 1–17). HAI treatment was stopped for progression, unacceptable toxicity, failure of HAI, or upon patient decision. More patients in the HAI-Folfirinox group received only a short course of HAI chemotherapy (< 4 infusions in 37.2% as compared to 29.6% in the HAI-ox group; Table 2).

**TABLE 2:** Treatment delivery.

Hepatic arterial infusion treatment	All patients *N* = 273	HAI-folfirinox *N* = 52	HAI-oxaliplatin *N* = 221	*p*
**Concomitant intravenous treatment**				
None	7 (2.6%)	5 (13.5%)	2 (0.9%)	<0.001***
Cetuximab/ panitumumab	79 (28.9%)	29 (78.4%)	50 (23.0%)	<0.001***
Bevacizumab	34 (12.5%)	3 (8.1%)	31 (14.3%)	0.01*
5FU	209 (76.6%)	0 (0.0%)	209 (96.3%)	0.001**
Irinotecan	78 (28.6%)	0 (0.0%)	78 (35.9%)	0.318 ns
Oxaliplatin	5 (1.8%)	0 (0.0%)	5 (2.3%)	0.083 ns
Raltitrexed	3 (1.1%)	0 (0.0%)	3 (1.4%)	0.002*
Trifluridine-tipiracil	1 (0.4%)	0 (0.0%)	1 (0.5%)	<0.001***
Unknown	19	15	4	
**Number of HAI infusions**				
<4	82 (31.1%)	19 (37.2%)	63 (29.6%)	0.032*
4–8	130 (49.2%)	21 (41.2%)	109 (51.2%)	0.063 ns
≥9	52 (19.7%)	11 (21.6%)	41 (19.2%)	0.955 ns
Unknown	9	1	8	

Concomitant intravenous treatments and intra-arterial treatment duration. *p < 0.05; **p < 0.01; and ***p < 0.001.

Concomitant IV cytotoxic chemotherapy was administered in 96.3% of patients in the HAI-Ox group and in no patients in the HAI-Folfirinox group. IV-targeted therapies were more frequently administered with HAI-Folfirinox (86.5%) than with HAI-Ox (37.3%), in particular for anti-epidermal growth factor receptor (EGFR; Table 2).

### Efficacy

**FIGURE 1:**
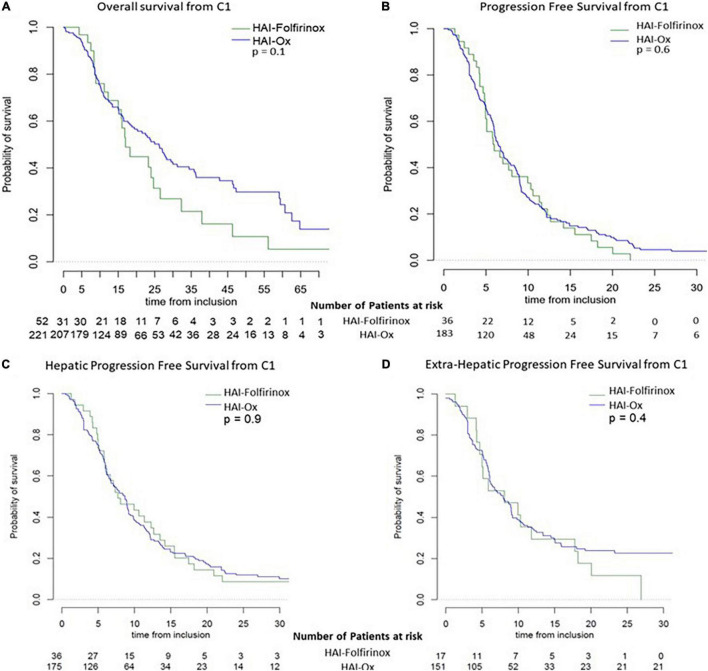
Survival curves depend on HAI treatment. Overall survival **(A)** and progression-free survival **(B)** in patients with HAI-Folfirinox and HAI-Ox. Specific hepatic progression-free survival **(C)** and extra-hepatic progression-free survival **(D)** in patients with HAI-Folfirinox and HAI-Ox.

Considering the overall population treated with HAI in this cohort, 45.4% of patients presented an objective response, i.e., 11.5% with a complete response, 32.4% presented stable disease, and 22.2% presented primary progressive disease, leading to a disease control rate of 77.8% (Table 3). With a median follow-up of 30.6 months (95% CI: 21.7–39.9), median OS was 23.9 months (95% CI: 18.2–28.2). Median global PFS and hepatic PFS were 6.4 months (95% CI: 6.0–7.6) and 7.9 months (95% CI: 6.5–9.0), respectively (Figures 1A,B). HAI-Folfirinox, as compared to HAI-Ox, neither improve OS (median 17.0 months (95% CI: 15.0–32.3) vs. 26.2 months (95% CI: 19.4–34.4, *p* = 0.1) nor median PFS 5.9 months (95% CI: 4.9–10.3) vs. 6.4 months (95% CI: 6.0–7.7, *p* = 0.6), respectively.

**TABLE 3:** Treatment efficacy: best response rates.

Type of HAI		All lines of treatment			Second line of treatment			Third and further lines of treatment		
		All *N* = 273	FOLFIRINOX *N* = 52	OX *N* = 221	All *N* = 116	FOLFIRINOX *N* = 24	OX *N* = 92	All *N* = 157	FOLFIRINOX *N* = 28	OX *N* = 129
Objective response rate	Yes	102 (45.4%)	19 (43.2%)	83 (45.9%)	51 (52.6%)	9 (40.9%)	42 (56.0%)	53 (39.3%)	10 (45.5%)	43 (38.1%)
	No	123 (54.7%)	25 (56.8%)	98 (54.1%)	46 (47.4%)	13 (59.1%)	33 (44.0%)	82 (60.7%)	12 (54.5%)	70 (61.9%)
	NA	48	8	40	20	2	17	22	6	15
Disease control rate	Yes	175 (77.8%)	33 (75.0%)	142 (78.5%)	80 (82.5%)	18 (81.8%)	62 (82.7%)	98 (39.0%)	15 (68.2%)	83 (73.5%)
	No	50 (22.2%)	11 (25.0%)	39 (21.5%)	17 (17.5%)	4 (18.2%)	13 (17.3%)	37 (60.3%)	7 (31.8%)	30 (26.5%)
	NA	48	8	40	19	2	17	22	6	16
Secondary hepatic resection	Yes	50 (20.1%)	16 (35.6%)*	34 (16.7%)*	31 (29.8%)	9 (40.9%)	22 (26.8%)	21 (14.2%)	7 (30.4%)	14 (11.2%)
	No	199 (79.9%)	29 (64.4%)	170 (83.3%)	73 (70.2%)	13 (59.1%)	60 (73.2%)	127 (85.8%)	16 (69.6%)	111 (88.8%)
	NA	24	7	17	13	2	11	11	5	6

HAI, hepatic arterial infusion. *p < 0.05.

No significant differences were observed in hepatic recurrences or in extra-hepatic recurrences depending on the type of HAI received (Figures 1C,D). Median extra-hepatic PFS was 8.1 months (95% CI: 5.0–18.2) and 7.9 months (95% CI: 6.1–9.2; *p* = 0.4) in patients treated with HAI-Folfirinox and HAI-Ox, respectively. Median hepatic-specific PFS was 7.7 months (95% CI: 6.0–13.5) and 8.7 months (95% CI: 6.8–9.2; *p* = 0.9) in patients treated with HAI-Folfirinox and HAI-Ox, respectively.

In the whole population, previous exposure to oxaliplatin led to similar median OS (23.6 months (95% CI: 17.7–31.2) vs. 23.9 months (95% CI: 16.0–46.3) without previous exposure to oxaliplatin; *p* = 0.7) and PFS 6.1 months (95% CI: 5.8–7.1) vs. 9.1 months (95% CI: 6.21–10.6) respectively; *p* = 0.9).

When looking at post-HAI treatments: 20.0% had a secondary hepatic resection and 12.8% had a secondary radiofrequency ablation. Secondary liver surgery was performed in 35.6% of patients treated with HAI-Folfirinox and 16.7% of patients treated with HAI-Ox (*p* = 0.007). The rate of secondary radiofrequency ablation was similar in the HAI-Folfirinox (16.7%) and HAI-Ox (12.4%, *p* = 0.7) groups.

**FIGURE 2:**
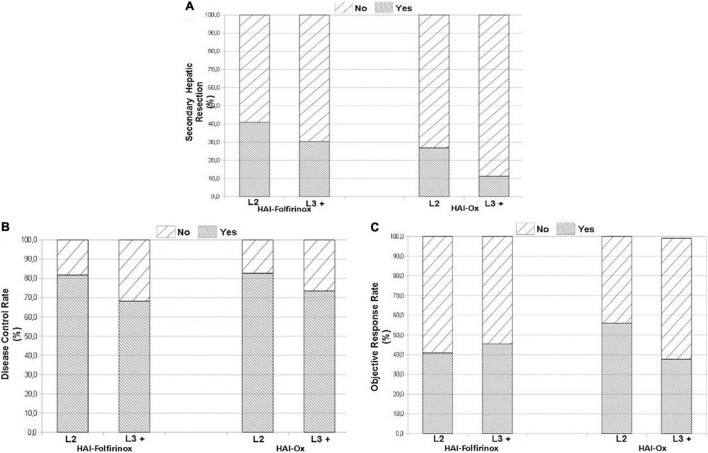
Treatment efficacy when administered second line (L2) or in further lines of treatment (L3 +). Secondary hepatic resection of metastases after hepatic arterial infusion (HAI) treatment **(A)**. Disease control rate **(B)**. Objective response rate **(C)**.

The use of HAI-Ox in second-line treatment as compared to later lines (Table 3) resulted in non-significant trends in secondary liver resections (26.8% vs. 11.2%; *p* = 0.12; Figure 2A), disease control rate (82.5% vs. 73.5%, *p* = 0.50; Figure 2B), and objective response rate (ORR; 56.0% vs. 38.1%, *p* = 0.21; Figure 2C). Similar trends were observed with HAI-Folfirinox. Patients on HAI chemotherapy had a median OS of 15.9 months (range 0.3–96.3) in second-line treatment and 11.2 months (range 0.6–70.9) beyond second line.

### Tolerability

During treatment, a dose reduction was performed in 62.5% of patients in the HAI-Folfirinox group and in 34.1% in the HAI-Ox group (*p* = 0.001). Before HAI treatment, 91% of patients presented a grade of 0 or 1 neuropathy. During treatment, grade ≥ 2 neuropathy appeared in 20.4% of patients in the HAI-Folfirinox group and in 29.3% of patients in the HAI-Ox group (ns).

Vascular complications occurred in 21.0% of all HAI-treated patients and local complications (skin necrosis, hematoma, healing failure, and local infection) occurred in 23.8% of patients. Local complications were significantly more frequent in patients with HAI-Folfirinox (34.9% vs. 17.9%, *p* = 0.01). Grade ≥ 2 neutropenia (42.5% vs. 22.7%; *p* < 0.001), diarrhea (34.9% vs. 13.9%; *p* = 0.005), and vomiting (20.0% vs. 6.0%; *p* = 0.006) were also more frequent in patients with HAI-Folfirinox when compared with patients with HAI-Ox. Vascular complications included hepatic artery occlusion, catheter occlusion, arteritis, and shunts requiring radiologic embolization of extra-hepatic arteries, such as the gastroduodenal artery even without any ulcer diagnosed. Among vascular complications, hepatic artery occlusion was reported four times, each time in patients receiving HAI-Folfirinox. One toxic death occurred in each group (Supplementary Table 2).

### Prognostic Factors in All Patients on Hepatic Arterial Infusion Treatments

In univariable analysis, parameters associated with poor OS were WHO-PS 2-3, peritoneal metastases, previous administration of irinotecan, bevacizumab treatment before HAI treatment, more than two previous treatment lines, and local complications related to the HAI catheter. Parameters associated with prolonged OS were liver metastases only and secondary hepatic resection (Supplementary Table 3).

In multivariable analysis, only 2 factors were associated with OS (Supplementary Table 3): the liver-only disease was associated with a prolonged OS (HR: 0.40; 95% CI 0.20–0.83; *p* = 0.013) and local complications related to HAI administration of the treatment were associated with a reduced OS (HR: 3.8; 95% CI 1.60–9.02; *p* = 0.002).

## Discussion

This study retrospectively compared HAI-Folfirinox to HAI-Ox in 273 patients with non-resectable liver metastases of colorectal cancer. Treatment was feasible and well tolerated in these patients with disease refractory to at least one line of treatment. Most of them had previously received IV oxaliplatin, irinotecan, and targeted therapies (bevacizumab or cetuximab). Treatment duration, PFS, and OS were similar between the two treatment modalities. HAI-Folfirinox was associated with significantly more secondary hepatic resections and more local complications linked to the catheter and treatment-related grade ≥ 2 toxicities, such as neutropenia, diarrhea, and vomiting. In multivariable analysis in the whole population receiving HAI, liver limited disease was associated with a prolonged OS whereas local complications were associated with shorter OS.

### Hepatic Arterial Infusion Results in Longer Overall Survival

In our study, patients on HAI chemotherapy had a median OS of 15.9 months in second-line treatment and 11.2 months beyond the second line, as compared to reported OS of 13 months (13, 14) and 6 months, respectively, for IV treatment in the literature (15, 16). As for the ORR, we report a lower ORR (45.3%) than Ducreux et al. (6) (64%; 95% CI, 44–81%) and Boige et al. (9) (55%; 95% CI, 40–69%). In both studies, patients in second-line therapy were treated with HAI-Ox combined with IV 5-FU with a median of 8 or 9 cycles received. In Ducreux et al., they had not previously received oxaliplatin. It is worth noting that we analyzed a much larger population of non-selected patients with the extra-hepatic disease (30.5%), pretreated with targeted therapies, and cytotoxic chemotherapies, which may account for the lower ORR than expected. Altogether, our results promote HAI as an interesting option in LMCRC beyond first-line treatment. HAI treatment appeared well tolerated, especially when administered with oxaliplatin only. A randomized trial specifically designed to compare HAI treatment to IV chemotherapy in patients with non-resectable LMCRC after induction treatment is currently ongoing (17).

### Hepatic Arterial Infusion to Increase Secondary Resection Rate

The use of HAI-Folfirinox relied on the resection rate of 16.7% and reported for patients treated with HAI-Folfirinox after two or three lines of treatment in the prospective OPTILIV trial (11). In terms of secondary resection, we noted a significantly higher secondary resection rate in patients with HAI-Folfirinox (35.6%). Our population included 42.9% of patients in a second-line setting, but the difference in secondary resection rate achieved was even greater between HAI-Folfirinox and HAI-Ox in third and further lines when compared with the second-line setting. Patients in the HAI-Ox group more often had extra-hepatic lesions (33.0% vs. 19.6%). The most frequent extra-hepatic lesions reported were lung metastases, which are less life threatening than peritoneal metastases. The peritoneal disease is rarely reported in this series: 0% in the HAI-Folfirinox group and 6.2% in the HAI-Ox group. The low number of patients concerned, resulting in a lack of statistical power, may account for the non-significance of the differences observed. In contrast with the OPTILIV trial, secondary surgical resection did not emerge as a predictor of longer OS in this work. HAI-Folfirinox induced more toxicities than HAI-Ox and more than one-third of patients received less than 2 months of treatment. HAI-Folfirinox did not significantly improve median OS as compared to HAI-Ox. These results suggest that resection of hepatic metastases should not be the predominant parameter used to guide treatment decision after second-line treatment. The increasing life expectancy of patients with LMCRC justifies randomized trials with HAI to conclude with certainty that the resection rate correlates with OS (OSCAR NCT02885753, SULTAN NCT03164655).

### Hepatic Arterial Infusion and Other Local Treatments

Other local treatments, such as chemoembolization with drug-eluting beads loaded with irinotecan (DEBIRI) (18) or selective internal radiation therapy (SIRT) (19), showed better OS than IV chemotherapy in patients pretreated for LMCRC. First attempts at comparing SIRT and HAI chemotherapy to set up the best treatment sequences have been published. They ruled in favor of HAI chemotherapy, which appeared as a predictor of longer OS (31.2 vs. 16.3 months; *p* < 0.001) when compared to SIRT (20). In addition, HAI is technically difficult after internal SIRT, which may affect the liver vasculature (21). Therefore, there is a need for HAI chemotherapy optimization. In this regard, previous reports suggest that several lines of HAI chemotherapy could be proposed before switching to another therapeutic strategy (22).

### Limits and Strengths

The retrospective and non-randomized nature of our study limits the strength of the conclusions that can be drawn from this work. Especially, the period of inclusion from 2008 to 2019 accounts for a significant percentage of missing data in laboratory analyses. *BRAF* and *RAS* testing in France was recommended in 2014 and implemented in 2015. We were therefore not able to adjust data for *RAS* and *BRAF* status. In addition, the heterogeneity of previous lines of treatment and concomitant treatments to HAI may have introduced bias. Especially, the concomitant administration of cetuximab was unbalanced between the HAI-Folfirinox and HAI-oxaliplatin groups (78.4% vs. 23%, respectively). Although one of the largest series reported in HAI-treated patients, it remained too small for efficient application of a propensity score. These results show the importance of conducting randomized prospective studies in the field, which is the only method to build comparable treatment groups and determine the best HAI modalities.

Nevertheless, we report here, to our knowledge, the largest series of HAI-treated patients after failure of a first-line IV chemotherapy and the first series comparing two HAI regimens. As HAI chemotherapy is of particular interest in achieving resection of liver metastases (23), this study provides evidence to help digestive oncologists and surgeons to adapt the modality of HAI treatment to their care objectives.

## Conclusion

Hepatic arterial infusion treatment is an effective option in patients with LMCRC refractory to previous lines of treatments. Intensifying HAI treatment led to a higher rate of secondary hepatic resection with no significant impact on OS and PFS. Our study suggests that when resection is the main objective of the treatment, intensification of HAI can be proposed for selected patients.

## Data Availability Statement

The data that support the findings of this study are available from the corresponding author, upon reasonable request.

## Ethics Statement

Ethical review and approval was not required for the study on human participants in accordance with the local legislation and institutional requirements. Written informed consent for participation was not required for this study in accordance with the national legislation and the institutional requirements.

## Author Contributions

VR completed the database, wrote, and revised the manuscript. SP initiated the project and the database. BS, DS, AL, YT, CG, AT, SJ, RG, PR, and MK reported cases to the study. EA performed the statistical analyses. JT initiated the study and supervised the project and the writing. All authors contributed to the article and approved the submitted version.

## Conflict of Interest

The authors declare that the research was conducted in the absence of any commercial or financial relationships that could be construed as a potential conflict of interest.

## Publisher’s Note

All claims expressed in this article are solely those of the authors and do not necessarily represent those of their affiliated organizations, or those of the publisher, the editors and the reviewers. Any product that may be evaluated in this article, or claim that may be made by its manufacturer, is not guaranteed or endorsed by the publisher.

## References

[1] Fong Y Cohen AM Fortner JG Enker WE Turnbull AD Coit DG Liver resection for colorectal metastases. *J Clin Oncol.* (1997) 15:938–46. 10.1200/JCO.1997.15.3.938 9060531

[2] Van Cutsem E Nordlinger B Adam R Köhne CH Pozzo C Poston G Towards a pan-European consensus on the treatment of patients with colorectal liver metastases. *Eur J Cancer.* (2006) 42:2212–21. 10.1016/j.ejca.2006.04.012 16904315

[3] Kennedy AS McNeillie P Dezarn WA Nutting C Sangro B Wertman D Treatment parameters and outcome in 680 treatments of internal radiation with resin 90Y-microspheres for unresectable hepatic tumors. *Int J Radiat Oncol Biol Phys.* (2009) 74:1494–500. 10.1016/j.ijrobp.2008.10.005 19157721

[4] Jashodeep D Narayan RR Kemeny NE D’Angelica MI. Role of hepatic artery infusion chemotherapy in treatment of initially unresectable colorectal liver metastases: a review. *JAMA Surg.* (2019) 154:768–76. 31188415 10.1001/jamasurg.2019.1694

[5] Tanaka T Arai Y Inaba Y Matsueda K Aramaki T Takeuchi Y Radiologic placement of side-hole catheter with tip fixation for hepatic arterial infusion chemotherapy. *J Vasc Interv Radiol.* (2003) 14:63–8. 10.1097/01.rvi.0000052292.26939.59 12525587

[6] Ducreux M Ychou M Laplanche A Gamelin E Lasser P Husseini F Hepatic arterial oxaliplatin infusion plus intravenous chemotherapy in colorectal cancer with inoperable hepatic metastases: a trial of the gastrointestinal group of the federation Nationale des centres de lutte contre le Cancer. *J Clin Oncol.* (2005) 23:4881–7. 10.1200/JCO.2005.05.120 16009952

[7] Goéré D Deshaies I de Baere T Boige V Malka D Dumont F Prolonged survival of initially unresectable hepatic colorectal cancer patients treated with hepatic arterial infusion of oxaliplatin followed by radical surgery of metastases. *Ann Surg.* (2010) 251:686–91. 10.1097/SLA.0b013e3181d35983 20224373

[8] Goéré D Benhaim L Bonnet S Malka D Faron M Elias D Adjuvant chemotherapy after resection of colorectal liver metastases in patients at high risk of hepatic recurrence: a comparative study between hepatic arterial infusion of oxaliplatin and modern systemic chemotherapy. *Ann Surg.* (2013) 257:114–20. 10.1097/SLA.0b013e31827b9005 23235397

[9] Boige V Malka D Elias D Castaing M De Baere D Goere D Hepatic arterial infusion of oxaliplatin and intravenous LV5FU2 in unresectable liver metastases from colorectal cancer after systemic chemotherapy failure. *Ann Surg Oncol.* (2008) 15:219–26. 10.1245/s10434-007-9581-7 17896145

[10] Goéré D Pignon JP Gelli M Elias D Benhaim L Deschamps F Postoperative hepatic arterial chemotherapy in high-risk patients as adjuvant treatment after resection of colorectal liver metastases – a randomized phase II/III trial – PACHA-01 (NCT02494973). *BMC Cancer.* (2018) 18:787. 10.1186/s12885-018-4697-7 30081865 PMC6080555

[11] Lévi FA Boige V Hebbar M Smith D Lepère C Focan C Conversion to resection of liver metastases from colorectal cancer with hepatic artery infusion of combined chemotherapy and systemic cetuximab in multicenter trial OPTILIV. *Ann Oncol.* (2016) 27:267–74. 10.1093/annonc/mdv548 26578731

[12] Falcone A Ricci S Brunetti I Pfanner E Allegrini G Barbara C Phase III trial of infusional fluorouracil, leucovorin, oxaliplatin, and irinotecan (FOLFOXIRI) compared with infusional fluorouracil, leucovorin, and irinotecan (FOLFIRI) as first-line treatment for metastatic colorectal cancer: the gruppo oncologico nord ovest. *J Clin Oncol.* (2007) 25:1670–6. 10.1200/JCO.2006.09.0928 17470860

[13] Van Cutsem E Tabernero J Lakomy R Prenen H Prausová J Macarulla T Addition of aflibercept to fluorouracil, leucovorin, and irinotecan improves survival in a phase III randomized trial in patients with metastatic colorectal cancer previously treated with an oxaliplatin-based regimen. *J Clin Oncol.* (2012) 30:3499–506. 10.1200/JCO.2012.42.8201 22949147

[14] Bennouna J Sastre J Arnold D Österlund P Greil R Van Cutsem E Continuation of bevacizumab after first progression in metastatic colorectal cancer (ML18147): a randomised phase 3 trial. *Lancet Oncol.* (2013) 14:29–37. 10.1016/S1470-2045(12)70477-1 23168366

[15] Grothey A Van Cutsem E Sobrero A Siena S Falcone A Ychou M Regorafenib monotherapy for previously treated metastatic colorectal cancer (CORRECT): an international, multicentre, randomised, placebo-controlled, phase 3 trial. *Lancet.* (2013) 381:303–12. 10.1016/S0140-6736(12)61900-X 23177514

[16] Mayer RJ Van Cutsem E Falcone A Yoshino T Garcia-Carbonero R Mizunuma N Randomized trial of TAS-102 for refractory metastatic colorectal cancer. *N Engl J Med.* (2015) 372:1909–19. 10.1056/NEJMoa1414325 25970050

[17] Boilève A Maillard A Wagner M Dromain C Laurent C Dupont Bierre E Treatment intensification with hepatic arterial infusion chemotherapy in patients with liver-only colorectal metastases still unresectable after systemic induction chemotherapy – a randomized phase II study – SULTAN UCGI 30/PRODIGE 53 (NCT03164655)– study protocol. *BMC Cancer.* (2020) 20:74. 10.1186/s12885-020-6571-7 32000724 PMC6990591

[18] Fiorentini G Aliberti C Tilli M Mulazzani L Graziano F Giordani P Intra-arterial infusion of irinotecan-loaded drug-eluting beads (DEBIRI) versus intravenous therapy (FOLFIRI) for hepatic metastases from colorectal cancer: final results of a phase III study. *Anticancer Res.* (2012) 32:1387–95. 22493375

[19] Hendlisz A Van den Eynde M Peeters M Maleux G Lambert B Vannoote J Phase III trial comparing protracted intravenous fluorouracil infusion alone or with yttrium-90 resin microspheres radioembolization for liver-limited metastatic colorectal cancer refractory to standard chemotherapy. *J Clin Oncol.* (2010) 28:3687–94. 10.1200/JCO.2010.28.5643 20567019

[20] Dhir M Zenati MS Jones HL Bartlett DL Choudry MHA Pingpank JF Effectiveness of hepatic artery infusion (HAI) versus selective internal radiation therapy (Y90) for pretreated isolated unresectable colorectal liver metastases (IU-CRCLM). *Ann Surg Oncol.* (2018) 25:550–7. 10.1245/s10434-017-6265-9 29181682

[21] Pernot S Velut G Hampig Kourie R Amouyal G Sapoval M Pointet AL 5-FU or mitomycin C hepatic arterial infusion after failure of arterial oxaliplatin in patients with colorectal cancer unresectable liver metastases. *Clin Res Hepatol Gastroenterol.* (2017) 42:255–60. 10.1016/j.clinre.2017.11.004 29233520

[22] Datta J Narayan RR Kemeny NE D’Angelica MI. Role of hepatic artery infusion chemotherapy in treatment of initially unresectable colorectal liver metastases: a review. *JAMA Surg.* (2019) 154:768–76. 10.1001/jamasurg.2019.1694 31188415

[23] Kehm RD Yang W Tehranifar P Terry MB. 40 years of change in age- and stage-specific cancer incidence rates in US women and men. *JNCI Cancer Spectr.* (2019) 3:kz038. 10.1093/jncics/pkz038 31414075 PMC6686848

