# Supplementary material for: Hepatic Arterial Infusion Chemotherapy With Folfirinox or Oxaliplatin Alone in Metastatic Colorectal Cancer
Source: Front Med (Lausanne). 2022 Jun 16;9:830595. doi: 10.3389/fmed.2022.830595 (PMC9243466; doi:10.3389/fmed.2022.830595)
Supplement: Supplementary file 1 [file Table_1.DOCX]

**Supplementary Table 1. Population Characteristics.** HAI: Hepatic Arterial Infusion

| **HAI treatment** | **All patients** | **HAI-Folfirinox** | **HAI-Ox** | **p** |
| --- | --- | --- | --- | --- |
|  | N = 273 | N = 52 | N = 221 |  |
| **Sex** |  |  |  | **0.578** |
| Female | 108 (39.7%) | 22 (43.1%) | 86 (38.9%) |  |
| Male | 164 (60.3%) | 29 (56.9%) | 135 (61.1%) |  |
| **Age** |  |  |  | **0.085** |
| <65 | 184 (68.9%) | 41 (78.9%) | 143 (66.5%) |  |
| >65 | 83 (31.1%) | 11 (21.1%) | 72 (33.5%) |  |
| Unknown | 6 | 0 | 6 |  |
| **Resected Primary Tumour** |  |  |  | **0.582** |
| Yes | 61 (22.5%) | 10 (19.6%) | 51 (23.2%) |  |
| No | 210 (77.5%) | 41 (80.4%) | 169 (76.8%) |  |
| Unknown | 2 | 1 | 1 |  |
| **Liver Metastases only** |  |  |  | **0.061** |
| Yes | 189 (69.5%) | 41 (80.4%) | 148 (67.0%) |  |
| No | 83 (30.5%) | 10 (19.6%) | 73 (33.0%) |  |
| Unknown | 1 | 1 | 0 |  |
| **Line of treatment** |  |  |  | **0.812** |
| 2 | 117 (42.9%) | 24 (46.2%) | 93 (42.1%) |  |
| 3 | 88 (32.2%) | 14 (26.9%) | 74 (33.5%) |  |
| 4 | 48 (17.6%) | 10 (19.2%) | 38 (17.2%) |  |
| 5+ | 20 (7.3%) | 4 (7.7%) | 16 (7.2%) |  |
| **WHO Performans status** |  |  |  | **0.005** |
| 0 | 90 (40.1%) | 29 (59.2%) | 64 (35.0%) |  |
| 1 | 128 (56.0%) | 19 (38.8%) | 111 (60.7%) |  |
| 2 | 7 (3.0%) | 0 (0%) | 7 (3.8%) |  |
| 3 | 2(0.9%) | 1 (2.0%) | 1 (0.5%) |  |
| Unknown | 41 | 3 | 38 |  |
| **Previous treatments** |  |  |  |  |
| **Oxaliplatin** |  |  |  | **0.008** |
| Yes | 231 (84.9%) | 38 (73.1%) | 193 (87.7%) |  |
| Unknown | 1 | 0 | 1 |  |
| **Irinotecan** |  |  |  | **0.428** |
| Yes | 231 (84.9%) | 46 (88.5%) | 185 (84.1%) |  |
| Unknown | 1 | 0 | 1 |  |
| **Anti-EGF receptor** |  |  |  | **0.897** |
| Yes | 91 (33.5%) | 17 (67.3%) | 74 (33.6%) |  |
| Unknown | 1 | 0 | 1 |  |
| **Anti-VEGF** |  |  |  | **0.004** |
| Yes | 173 (63.6%) | 42 (80.8%) | 131 (59.6%) |  |
| Unknown | 1 | 0 | 1 |  |
| **Trifluridine-Tipiracil** |  |  |  | **1** |
| Yes | 2 (0.7%) | 0 (0%) | 2 (1.0%) |  |
| Unknown | 1 | 0 | 1 |  |
| **Regorafenib** |  |  |  | **0.587** |
| Yes | 5 (1.8%) | 0 (0%) | 5 (2.3%) |  |
| Unknown | 1 | 0 | 1 |  |
