# Supplementary material for: Hepatic Arterial Infusion Chemotherapy With Folfirinox or Oxaliplatin Alone in Metastatic Colorectal Cancer
Source: Front Med (Lausanne). 2022 Jun 16;9:830595. doi: 10.3389/fmed.2022.830595 (PMC9243466; doi:10.3389/fmed.2022.830595)
Supplement: Supplementary file 2 [file Table_2.DOCX]

**Supplementary Table 2. Treatment toxicities.** HAI: Hepatic Arterial Infusion. Significant differencies are marked with *

| **HAI Treatment** | **All patients** | **HAI-Folfirinox** | **HAI-Ox** | **p** |
| --- | --- | --- | --- | --- |
|  | N = 273 | N = 52 | N = 221 |  |
| **Neutropenia** |  |  |  | **<0.001*** |
| < 2 | 183 (73.8%) | 22 (55.0%) | 141 (67.8%) |  |
| ≥ 2 | 65 (22.2%) | 18 (45.0%) | 47 (32.2%) |  |
| Unknown | 25 | 12 | 13 |  |
| **Anemia** |  |  |  | **0.004*** |
| < 2 | 229 (90.5%) | 37 (82.2%) | 192 (91.9%) |  |
| ≥ 2 | 24 (9.5%) | 8 (17.8%) | 16 (7.7%) |  |
| Unknown | 20 | 7 | 13 |  |
| **Thrombopenia** |  |  |  | **0.886** |
| **<2** | 221 (87.4%) | 38 (84.4%) | 183 (88.0%) |  |
| ≥ 2 | 32 (12.6%) | 7 (15.6%) | 25 (12.0%) |  |
| **Unknown** | 20 | 7 | 13 |  |
| **Abdominal Pain** |  |  |  | **0.071** |
| < 2 | 160 (72.1%) | 22 (55.0%) | 138 (75.8%) |  |
| ≥ 2 | 62 (27.9%) | 18 (45.0%) | 44 (24.2%) |  |
| Unknown | 51 | 12 | 39 |  |
| **Nausea** |  |  |  | **0.184** |
| < 2 | 185 (82.2%) | 31 (72.1%) | 154 (84.6%) |  |
| ≥ 2 | 40 (17.8%) | 12 (27.9%) | 28 (15.4%) |  |
| Unknown | 48 | 9 | 39 |  |
| **Vomiting** |  |  |  | **0.006 *** |
| < 2 | 207 (72.7%) | 36 (80.0%) | 171 (94.0%) |  |
| ≥ 2 | 20 (8.8%) | 9 (20.0%) | 11 (6.0%) |  |
| Unknown | 46 | 7 | 39 |  |
| **Diarrhea** |  |  |  | **0.005*** |
| < 2 | 207 (81.5%) | 30 (65.2%) | 177 (85.1%) |  |
| ≥ 2 | 47 (18.5%) | 16 (32.6%) | 31 (14.9%) |  |
| Unknown | 19 | 6 | 13 |  |
| **Neuropathy** |  |  |  | **0.465** |
| < 2 | 181 (71.8%) | 35 (79.5%) | 146 (70.2%) |  |
| ≥ 2 | 71 (28.2%) | 9 (20.5%) | 62 (29.8%) |  |
| Unknown | 21 | 8 | 13 |  |
| **Hepatic failure** |  |  |  | **0.126** |
| Yes | 39 (17.8%) | 11 (25%) | 28 (15.3%) |  |
| No | 188 (82.8%) | 33 (75%) | 155 (84.7%) |  |
| Unknown | 46 | 8 | 38 |  |
| **Local complications** |  |  |  | **0.15** |
| Yes | 54 (23.8%) | 6 (15%) | 48 (25.7%) |  |
| No | 173 (76.2%) | 34 (85%) | 139 (74.3%) |  |
| Unknown | 40 | 9 | 31 |  |
| **Vascular complications** |  |  |  | **0.014*** |
| Yes | 49 (21.0%) | 15 (34.9%) | 34 (17.9%) |  |
| No | 184 (79.0%) | 28 (65.1%) | 156 (82.1%) |  |
| Unknown | 40 | 9 | 31 |  |
| **Gastric/Duodenal Ulcer** |  |  |  | **0.47** |
| Yes | 10 (4.4%) | 2 (5%) | 8 (4.3%) |  |
| No | 215 (95.6%) | 38 (95%) | 177 (95.7%) |  |
| Unknown | 48 | 12 | 36 |  |
| **Toxic death** |  |  |  | **0.319** |
| Yes | 2 (0.9%) | 1 (2.6%) | 1 (0.5%) |  |
| No | 225 (99.1%) | 38 (97.4%) | 184 (99.5%) |  |
| Unknown | 49 | 13 | 36 |  |
