# Supplementary material for: Hepatic Arterial Infusion Chemotherapy With Folfirinox or Oxaliplatin Alone in Metastatic Colorectal Cancer
Source: Front Med (Lausanne). 2022 Jun 16;9:830595. doi: 10.3389/fmed.2022.830595 (PMC9243466; doi:10.3389/fmed.2022.830595)
Supplement: Supplementary file 3 [file Table_3.DOCX]

**Supplementary Table 3. Analysis of prognostic factors.** HR = Hazard Ratio. CI = confidence interval. Cox model; *: p<0.05. **: p<0.01. ***:p<0.001

|  | **Univariate Analysis** | | **Multivariate Analysis** | |
| --- | --- | --- | --- | --- |
|  | **HR [95%CI]** | **p** | **HR [95%CI]** | **p** |
| **Patient Characteristics** |  |  |  |  |
| Age >65 | 1.06 [0.72-1.562] | 0.768 |  |  |
| Male sex | 0.959 [0.665-1.383] | 0.823 |  |  |
| WHO performans status 2-3 | 5.592 [1.969-15.878] | 0.001** | 5.592 [0.242-3.885] | 0.965 |
| **Tumour characteristics** |  |  |  |  |
| Resected Primary Tumour | 0.949 [0.619-1.456] | 0.812 |  |  |
| No microsatellite instability | 26171897.145 [0-Inf] | 0.998 |  |  |
| Wild Type KRAS | 0.509 [0.069-3.759] | 0.508 |  |  |
| Wild Type RAS | 0.535 [0.273-1.047] | 0.068 |  |  |
| Wild Type BRAF | 0.581 [0.227-1.489] | 0.258 |  |  |
| **Disease extension** |  |  |  |  |
| Liver metastases only | 0.594 [0.41-0.86] | 0.006** | 0.38 [0.189-0.764] | 0.007** |
| All metastases | 1.018 [0.925-1.121] | 0.712 |  |  |
| 5 to 10 metastases | 1.552 [0.773-3.118] | 0.216 |  |  |
| >10 metastases | 1.004 [0.132-7.664] | 0.997 |  |  |
| Uncountable liver metastases | 1.413 [0.913-2.188] | 0.121 |  |  |
| Peritoneum extension | 2.999 [1.598-5.628] | 0.001** |  |  |
| Lung extension | 1.224 [0.774-1.935] | 0.388 |  |  |
| Lymph node extension | 1.98 [0.96-4.084] | 0.064 |  |  |
| **Previously treated by** |  |  |  |  |
| Oxaliplatine | 0.933 [0.587-1.482] | 0.768 |  |  |
| Irinotecan | 4.376 [2.081-9.203] | 0*** |  |  |
| Anti-EGF receptor | 1.009 [0.684-1.486] | 0.966 |  |  |
| Anti-VEGF receptor | 2.54 [1.694-3.806] | 0*** |  |  |
| Tas-102 | 9.838 [1.287-75.185] | 0.028* |  |  |
| Regorafenib | 8.414 [2.567-27.577] | 0*** |  |  |
| >2 lines of treatment | 1.625 [1.124-2.348] | 0.01* | 0.851 [0.418-1.73] | 0.655 |
| Line of HAI chemotherapy | 1.363 [0.729-2.55] | 0.332 |  |  |
| **Biological Parameters** |  |  |  |  |
| Bilirubin Day 0 | 1.016 [1.004-1.027] | 0.008** | 1.013 [0.991-1.035] | 0.24 |
| ACE Day 0 | 1 [1-1.001] | 1 |  |  |
| ACE Day 0 >200 | 2.009 [1.115-3.618] | 0.02 | 1.173 [0.505-2.721] | 0.711 |
| CA 19-9 Day 0 | 1 [1] | 0*** | 1 [1] | 0.209 |
| **Treatment delivered** |  |  |  |  |
| HAI Oxaliplatin only | 1.078 [0.711-1.635] | 0.722 |  |  |
| Dose reduction | 0.803 [0.513-1.257] | 0.338 |  |  |
| **Observed Adverse effect** |  |  |  |  |
| Local complications | 2.083 [1.302-3.334] | 0.002** | 4.28 [1.837-9.972] | 0.001** |
| Vascular complications | 1.148 [0.707-1.864] | 0.577 |  |  |
| Gastric or Duodenal Ulcer | 0.58 [0.213-1.581] | 0.287 |  |  |
| **Secondary treatment achieved** |  |  |  |  |
| Hepatic resection | 0.466 [0.278-0.783] | 0.004** | 0.71 [0.187-2.692] | 0.615 |
| Hepatic Radiofrequency | 0.418 [0.209-0.838] | 0.014 | 0.601 [0.174-2.082] | 0.422 |
